# Specific Gene Disruption in the Major Livestock Pests *Cochliomyia hominivorax* and *Lucilia cuprina* Using CRISPR/Cas9

**DOI:** 10.1534/g3.119.400544

**Published:** 2019-07-24

**Authors:** Daniel F. Paulo, Megan E. Williamson, Alex P. Arp, Fang Li, Agustin Sagel, Steven R. Skoda, Joel Sanchez-Gallego, Mario Vasquez, Gladys Quintero, Adalberto A. Pérez de León, Esther J. Belikoff, Ana M. L. Azeredo-Espin, W. Owen McMillan, Carolina Concha, Maxwell J. Scott

**Affiliations:** * Centre for Molecular Biology and Genetic Engineering, Department of Genetics, Evolution, Microbiology and Immunology, University of Campinas; † Laboratory of Ecological and Evolutionary Genomics, Smithsonian Tropical Research Institute, Gamboa, Panama; ‡ Department of Entomology and Plant Pathology, North Carolina State University, Raleigh NC; § USDA-ARS, Knipling-Bushland U.S. Livestock Insects Research Laboratory and Veterinary Pest Genomics Center, Kerrville TX, and; ** USDA-ARS, Knipling-Bushland U.S. Livestock Insects Research Laboratory and Veterinary Pest Genomics Center, Screwworm Research Site, Pacora, Panama

**Keywords:** Functional genomics, reverse genetics, myiasis, New World Screwworm fly, Australian Sheep Blowfly, gene drive, CRISPR/Cas9, *brown body*, *yellow*, *transformer*

## Abstract

*Cochliomyia hominivorax* and *Lucilia cuprina* are major pests of livestock. Their larvae infest warm-blooded vertebrates and feed on host’s tissues, resulting in severe industry losses. As they are serious pests, considerable effort has been made to develop genomic resources and functional tools aiming to improve their management and control. Here, we report a significant addition to the pool of genome manipulation tools through the establishment of efficient CRISPR/Cas9 protocols for the generation of directed and inheritable modifications in the genome of these flies. Site-directed mutations were introduced in the *C*. *hominivorax* and *L*. *cuprina yellow* genes (*ChY* and *LcY*) producing lightly pigmented adults. High rates of somatic mosaicism were induced when embryos were injected with Cas9 ribonucleoprotein complexes (RNPs) pre-assembled with guide RNAs (sgRNAs) at high concentrations. Adult flies carrying disrupted *yellow* alleles lacked normal pigmentation (*brown body* phenotype) and efficiently transmitted the mutated alleles to the subsequent generation, allowing the rapid creation of homozygous strains for reverse genetics of candidate loci. We next used our established CRISPR protocol to disrupt the *C*. *hominivorax transformer* gene (*Chtra*). Surviving females carrying mutations in the *Chtra* locus developed mosaic phenotypes of transformed ovipositors with characteristics of male genitalia while exhibiting abnormal reproductive tissues. The CRISPR protocol described here is a significant improvement on the existing toolkit of molecular methods in calliphorids. Our results also suggest that Cas9-based systems targeting *Chtra* and *Lctra* could be an effective means for controlling natural populations of these important pests.

The New World Screwworm fly, *Cochliomyia hominivorax*, is a major livestock pest and the only obligatory ectoparasitic blowfly in the Neotropical region (Alexander 2006). Likewise, the Australian sheep blowfly, *Lucilia cuprina*, is the main species involved in primary flystrike in Australia and New Zealand (Sandeman *et al.* 2014). Adult females of these species are attracted to oviposit by odors emanated by their hosts (Zhu *et al.* 2017; Yan *et al.* 2018). After hatching, their larvae (maggots) infest and feed on host’s living flesh to complete development, which can ultimately lead to lethality if untreated. These infestations (known as myiasis) are responsible for severe economic impacts to the livestock industry, estimated in hundreds of millions spent annually in treatment, prevention and animal welfare (Alexander 2006; Grisi *et al.* 2014). Over the last 60-years, the Sterile Insect Technique (SIT) program has been successfully used to eradicate *C*. *hominivorax* from all North and Central America (see review by Scott *et al.* (2017)). Currently, the mass rearing biosecurity facility, at the Panama-United States Commission for the Eradication and Prevention of Screwworm (COPEG), is responsible for the dispersal of millions of factory-grown radiated-sterile screwworm males and females in the Darien barrier zone, along the Panama-Colombian border. The main goal is to prevent re-introductions of endemic flies from South America, where infestations remain a serious problem. With the goal of genetic control of *L. cuprina*, a “*field-female killing system*” was developed that showed promise in a field test (Foster *et al.* 1991) but the strain was never used for fly control due to instability during mass rearing. Consequently, the industry primarily relies on insecticides and good farming practices for control of *L. cuprina*. However, insecticide resistant alleles were rapidly selected, resulting in the near extinction of the blowfly’s susceptive genotype (see reviews by Sandeman *et al.* (2014), and Anstead *et al.* (2016)). Indeed, *L. cuprina* has shown resistance against nearly all classes of currently available insecticides, in perhaps the finest “*Chasing the Red Queen*” example, hypothesizing that it is just a matter of time before resistance against currently used chemicals emerges.

More recently, species-specific genetic control strategies for population suppression and/or the introduction of novel harm-reducing genetic traits in wild populations, became a promising alternative to the traditional pest control methods (Alphey and Bonsall 2018). These strategies also provide major improvements on the effectiveness of SIT, for instance, through the establishment of Transgenic-Sexing Strains (TSS), which carry a conditional female-specific lethal transgene into the species genome (Thomas *et al.* 2000; Heinrich and Scott 2000; Scott *et al.* 2014a). As *C. hominivorax* and *L. cuprina* are high-consequence pests of livestock, considerable effort has been made to develop genomic resources and functional genomic tools (Concha and Scott 2009; Li *et al.* 2013, 2014; Anstead *et al.* 2015; Yan and Scott 2015; Concha *et al.* 2016). However, although Cas9 was recently used to create a single knock-in mutation in the *L. cuprina no blokes* (*nbl*) gene (Davis *et al.* 2018), routinely successful methods for direct gene disruption or genome editing in calliphorids have not been developed, until now. The lack of an established tool capable of generating specific inheritable genomic modifications, that allow deeper investigations on gene functions, represents a barrier in dissecting the cellular and the molecular basis that underlies the biology and physiology of the parasitic traits of *C*. *hominivorax* and *L. cuprina*.

To overcome this significant constraint, we developed a highly efficient site-specific mutagenesis protocol for the generation of *C*. *hominivorax* and *L*. *cuprina* knockouts using the Clustered Regularly Interspaced Short Palindromic Repeats (CRISPR) and the CRISPR-associated protein 9 (Cas9) genome editing technology (CRISPR/Cas9; Doudna and Charpentier 2014). Cas9 is a DNA endonuclease enzyme capable of assembling ribonucleoprotein complexes (RNPs) with small (∼20 bp) single-stranded guide RNAs (sgRNAs) in laboratory conditions. When introduced into cells, these RNPs are guided to a specific genomic region by base pair complementary between the sgRNA and its target DNA sequence, which is directly followed by a protospacer adjacent motif (PAM, usually NGG-3′). Once the target is recognized, the sgRNA-guided Cas9 binds to its sequence and promotes double-strand breaks (DSBs) that will be preferentially repaired by the non-homologous end joining (NHEJ) pathway. As a consequence, this error-prone process commonly results in the introduction of deletions and/or insertions (collectively called indels) at the break site, leading to mutations which - *in protein-coding exons* - are typically premature stop codons or frameshifts that disrupt the peptide sequence and so the protein function (Carroll 2014). Due to its simplicity, efficiency and precision, CRISPR arises as a powerful genome editing technology, being successfully used to study gene function *in vivo* in a diversity of insects and other arthropods (see review by Sun *et al.* (2017)). Typically, research projects target genes linked to evident phenotypes (Bassett *et al.* 2013; Li and Scott 2016; Khan *et al.* 2017; Li *et al.* 2017b, 2017a; Heinze *et al.* 2017; Meccariello *et al.* 2017; Perera *et al.* 2018; Choo *et al.* 2018; Aumann *et al.* 2018; Liu *et al.* 2019; Sim *et al.* 2019, to cite some examples), which allows a rapid and unambiguous screening for successful Cas9-mediated editing events. In addition to the sequenced genome of *L. cuprina* (Anstead *et al.* 2015, 2016) and the current efforts to assemble and annotate the *C. hominivorax* genome (M.J. Scott, unpublished; A.C.M. Junqueira, unpublished), we believe that the establishment of the CRISPR technique will be a significant improvement on the existing toolkit of molecular methods in these species. Further, the technology offers the opportunity to engage in studies on novel, environmentally sustainable pest control technologies based on genome editing.

## Materials and Methods

### Fly rearing and mutant strain establishment

The wildtype (wt) strains J06, of *C*. *hominivorax*, and LA07, of *L*. *cuprina*, were used for this study. Screwworm flies were maintained in the USDA-ARS research site located within the COPEG biosecurity plant in Panama (life cycle showed in Figure S1), while blowflies were maintained in the insectary at the North Carolina State University, Raleigh (NC State) under conditions previously described by Concha *et al.* (2016) and Li *et al.* (2014), respectively. Microinjections were carried out in the same laboratory where each species was being maintained. A *C*. *hominivorax* strain carrying biallelic mutations at the *yellow* locus (*ChY*) was obtained by randomly crossing single G_2_ brown* body* (*bwb*; see results) males with J06 virgin females. The G_3_ offspring were let to inbreed freely in cages and all the obtained flies at G_4_ showing *bwb* phenotype were selected and interbred to establish the heteroallelic mutant strain *ChYellow*^07/01^ (see crossing scheme in Figure S2). *L. cuprina* strains homozygous for a single mutation at the *LcY* locus were obtained by crossing single G_1_ males with LA07 virgin females. Six single G_2_ males were then crossed with LA07 virgin females and their offspring allowed to interbred freely. Any G_4_ flies that showed the *bwb* phenotype were selected and interbred to establish strains homozygous for a single mutation at the *LcY* locus (crossing scheme showed in Figure S3). Molecular genotyping confirmed that these six strains were homozygous for two different mutations. Consequently, two strains with the different mutations (LcY1B and LcY1C) were maintained.

### sgRNA and Cas9

Single guide RNAs (sgRNAs) were designed by examining both DNA strands in the exons of each investigated gene for the presence of protospacer-adjacent motifs (PAM) with the sequence NGG-3′ (where N is any nucleotide) using the standalone version of CRISPOR tool (Concordet and Haeussler 2018). Considered sgRNAs sequences were 17-20 bp in length (excluding PAM) with *in silico* predicted minimal off-target effects on the genomes. Scanned sgRNAs containing GG before the PAM sequence (NGGNGG-3′), which may improve sgRNA efficacy (Farboud and Meyer 2015), and one or two 5′-end G at the start of the target sequence, which facilitates transcription by T7 RNA polymerase (Bassett and Liu 2014), were selected when possible. Replacement and additions of 5′-end guanines to the guide sequences were made when necessary. Synthesis of sgRNAs was performed as described for *Drosophila melanogaster* (Bassett and Liu 2014) with minor modifications. *C*. *hominivorax* templates were generated by *step-up* PCR in a 100 µl final volume containing 1x Phusion HF buffer, 200 µM of dNTPs, 2 U of Phusion DNA polymerase (NEB) and 0.5 µM of each CRISPR sgR-Specific-T7-FWD and sgR-Universal-REV primers. Amplification conditions included an initial denaturation step of 98° for 2 min, followed by 10 cycles of 98° for 10 sec, 60° for 30 sec and 72° for 15 sec. Annealing temperature was increased to 65° for the subsequent 25 cycles of amplification, followed by a final extension at 72° for 10 min. Expected 100 bp amplicons were confirmed by electrophoresis in 1x TAE (40 mM Tris-acetate, 1 mM EDTA) 2% agarose gels stained with GelRed, purified using the QIAquick PCR Purification Kit (Qiagen) and quantified with a Nanodrop 1000 spectrophotometer (ThermoFisher Scientific). *L. cuprina* templates were generated by PCR in 100 µl final volume containing 1x Q5 High-Fidelity 2x Master Mix (NEB) and 0.5 µM of each CRISPR primers (above). Amplification conditions included an initial denaturation step of 98° for 2 min, followed by 35 cycles of 98° for 10 sec, 58° for 10 sec, and 72° for 10 sec, followed by a final extension of 72° for 7 min. Expected 100 bp amplicons were confirmed by loading 5% of the PCR reaction on a 1X TBE 1% agarose gel stained with GelGreen. The remaining PCR was purified using the QIAquick PCR Purification Kit (Qiagen) and quantified with the Qubit HS-DNA Kit. *In vitro* transcription of the sgRNAs was carried out using the MEGAshortscript T7 Transcription Kit (Ambion) and 300 ng of DNA template for a final volume of 20 µl. Reactions were incubated at 37° for 4 h followed by TURBO DNase (Ambion) treatment using 2 U for a further 15 min at 37°. Transcriptions were extracted with Phenol:Chloroform:Isoamyl Alcohol (25:24:1, v/v, pH 6.7), precipitated with isopropanol and left overnight at -20°. RNA was collected by centrifugation, washed once with 85% ethanol and resuspended with RNAse-free ddH_2_O. Concentrations of the sgRNAs were measured using Nanodrop, adjusted to 2 µg/µl, aliquoted and stored at -80° until use. Cas9 mRNA was *in vitro* transcribed using the mMessage mMachine T7 Kit and polyadenylated using the Tailing Kit (Ambion). Recombinant Cas9 protein was obtained commercially (PNA Bio or NEB EnGen Cas9 NLS, *S. pyogenes*), diluted to 1 µg/µl in DNAse-free ddH_2_O, aliquoted and stored at -80° until use. Designed sgRNA can be found in the Supplemental Material, Table S1 in File S1.

### Microinjections

Ribonucleoprotein complexes (RNPs) were pre-assembled by incubating Cas9 protein (500 ng/µl or 360 ng/µl for *C. hominivorax*, and 483 ng/µl for *L. cuprina*) with sgRNA (200 ng/µl or 145 ng/µl) in a Sodium Phosphate Buffer (supplied with 300 mM of KCl to prevent Cas9 aggregation; Burger *et al.* 2016) at 37° for 10 - 30 min. For dual-targeting experiments, RNPs were pre-assembled using each sgRNA separately and then 1:1 mixed prior to the injections. To prevent needle clogging, the injection cocktail was centrifuged at top speed for 10 min at 4° and the upper portion spun through a 0.45 µm filter column (Millipore) and collected in a clean tube which was maintained on ice during the experiments. Needles were prepared in a P-2000 needle puller (Sutter Instrument) using quartz capillaries with filament (O.D.: 1.0 mm I.D.: 0.7, 10 cm length), and opened during injections by contact with the chorion of an embryo or beveled using a BV-10 Micropipette Beveler (Sutter Instrument). Adult *C. hominivorax* 6 d-old females were stimulated to lay eggs, which were then dissociated with KOH 4% (w/v) for 2 min and gently rinsed in ddH_2_O. Adult *L. cuprina* females were given fresh 93% ground beef to stimulate egg laying at 10 days old. Treated embryos were aligned on a double slide tape in concave well microscopy slides, dehydrated in a silicagel chamber for 3 - 10 min and then covered with Halocarbon 27 oil (Sigma). Cocktails were injected though the chorion membrane into the posterior end of pre-blastoderm embryos (less than 40 min old; Figure S1). Injections were performed using a XenoWorks Micromanipulator connected to a Digital Microinjector (Sutter Instrument) device set for a “continuous” injection mode. After injecting *C. hominivorax* embryos, excessive oil was carefully removed and injected embryos were rehydrated by ddH_2_O washing, while *L. cuprina* embryos remained covered under oil until hatching. Slides were placed in a petri dish containing humidified paper towel, and a small amount of larvae diet was placed surrounding the embryos. Plates were placed into a hyperbaric oxygen chamber and incubated overnight at 37° with 80% relative humidity for *C*. *hominivorax* embryos and 21 - 25° for *L. cuprina* embryos. Eclosing first instar G_0_ larvae were collected under white light using a Leica M165FC stereomicroscope, placed on diet and reared until adulthood, when mosaic adults were visually screened for the *bwb* mutation.

### Genotyping

Genomic DNA was extracted using the DNAreleasy Advance Direct Lysis Kit (Bulldog bio) or the DNAzol reagent (Invitrogen). PCR reactions were carried out for a final volume of 50 µl containing 1x Phusion HF buffer, 80 µM of dNTPs, 0.4 U of Phusion DNA polymerase (NEB) and 0.1 µM of each target specific forward and reverse primers. Samples were cycled under the following conditions: 98° for 2 min, 35 cycles of 98° for 10 sec, 60° for 30 sec and 72° for 30 sec, followed by a final extention step at 72° for 5 min. PCR products were verified by electrophoresis in a 1.5% agarose gel and purified as described above. Blunt-ended fragments were A-tailed with 1x PCR buffer, 200 µM of dATP, 5 U of *Taq* DNA Polymerase (QIAGEN) and 6 µl of previously purified PCR product. Reactions were incubated at 70° for 30 min and directly TA cloned into a pGEM-T Easy Vector (Promega). Plasmids were extracted using an alkaline lysis protocol and then digested with 20 U of *Eco*RI (NEB) at 37° for 2 h. Cloned fragments were verified by electrophoresis and submitted to Sanger sequencing in an ABI 3730xl DNA Analyzer (Applied Biosystems), using the BigDye Terminator Cycle Sequencing Kit (Applied Biosystems) with the universal primers M13-Forward and M13-Reverse. Raw reads were analyzed using FinchTV application (Geospiza Inc.) and assembled with CAP3 (Huang and Madan 1999). For Cas9 single target experiments, contigs were mapped against the reference sequence (wt) using the BWA-MEM algorithm (Li 2013; relaxed parameters used: -A 4 -O 2 -L 5 -B 4 -E 1 -U 10), and analyzed by CrispRVariants package v.1.8.0 (Lindsay *et al.* 2016). T7EN1 cleavage assays were performed by denaturing 10 µl of unpurified PCR product at 95° for 10 min in NEBBuffer #2 (NEB). Reactions were then incubated at room temperature for at least 1 h for amplicon hybridization. Heteroduplex DNA were digested with 2 U of T7 endonuclease I (NEB) at 37° for 20 min and the entire reaction volume resolved in a 3% agarose gel electrophoresis in cold 1x TBE buffer. Genotyping primers specifications can be found in Table S2.

### Data availability

The *C. hominivorax yellow* gene (*ChY*) was annotated from the current genome draft assembly (M.J. Scott, unpublished, genbank accession GCA_004302925.1) and it is available upon request, while *tra* gene (*Chtra*) was retrieved from GenBank (accession number JX315618). The *L. cuprina yellow* gene is available at NCBI RefSeq under the accession number XM_023449239. All additional information is available as Supplemental material available at FigShare: https://doi.org/10.25387/g3.8868578.

## Results

### Cas9-induced somatic and inheritable brown body mutation in the yellow locus of C. hominivorax

**Figure 1:**
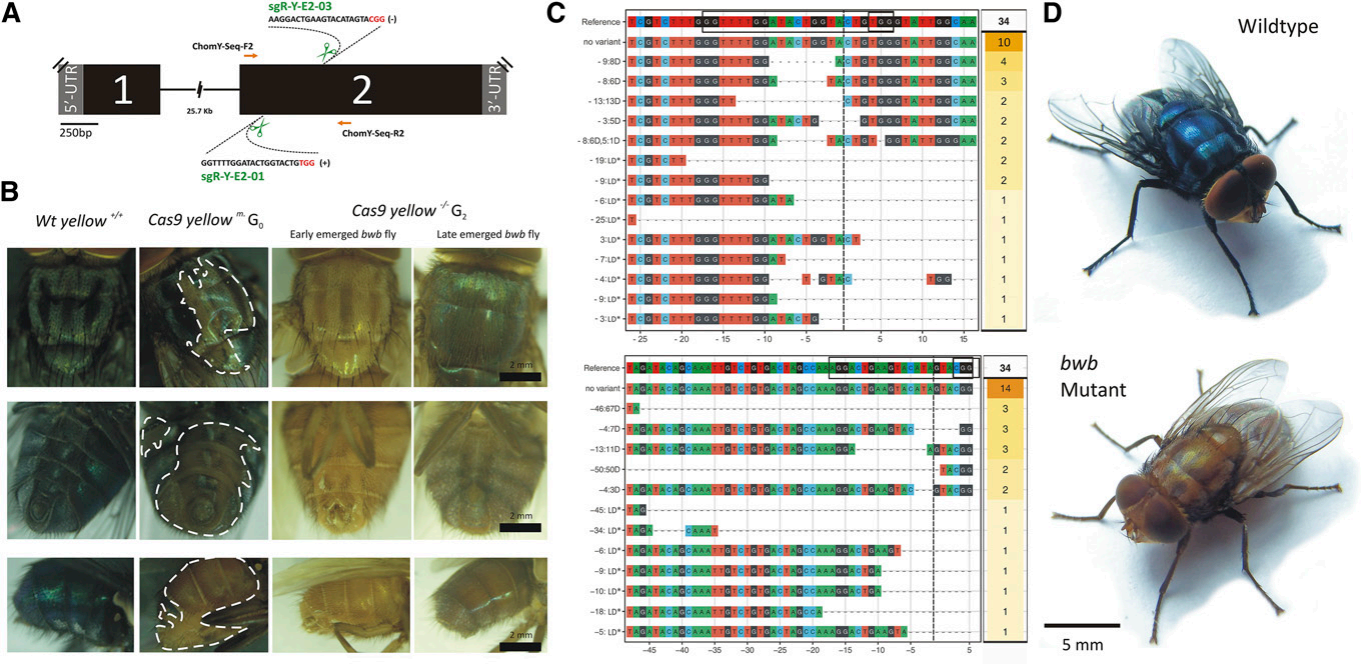
Cas9-mediated disruption of the *yellow* loci (*ChY*) promotes the brown* body* mutation (*bwb*) in the screwworm fly, *Cochliomyia hominivorax*. (A) Schematic of *ChY* genomic organization with exons as numbered black boxes, introns as lines and untranslated regions (UTRs) as gray boxes. Two sgRNAs were designed 368 bp apart to dual-target *ChY* exon 2. Cas9 RNPs targeted sites are indicated by scissors with their respectively sgRNA (PAM motifs are shown in red). Genotyping primers are indicated as orange arrows. (B) The *bwb*-mutant flies (leftmost) in comparison with the wildtype (wt) (rightmost) phenotype. The presence of yellowish body areas in the G_0_ flies reveals biallelic hits in the *ChY* loci (regions encircled with a white broken line). Knockout individuals (*yellow* ^−/−^) were obtained at G_2_. The *bwb*-mutant flies showed a partial recovery of the cuticle pigmentation some hours after the emergence. (C) Sequencing confirmation of target-specific disruption of the *ChY* loci at sgRNAs target sites. Alleles recovered from G_0_ founders were mapped against the wt reference sequence and analyzed using the CrispRVariants pipeline (Lindsay *et al.* 2016). Targeted sites and PAM motifs are boxed. Predicted Cas9 cut sites are indicated by vertical black dashed line. LD* indicates alleles with large deletions between both sgRNAs (see Figure S5 for a more comprehensive alignment). (D) Habitus (*i.e.*, general appearance) comparison between flies from the wt strain J06 (above, greenish-blue colored) and early emerged *bwb*-mutant strain *ChYellow*^07/01^ (bellow, metallic yellowish).

Aiming to demonstrate the potential of gene specific knockout in the screwworm fly using the CRISPR/Cas9 technology, we selected *yellow* (*ChY*) as our candidate target gene. The *ChY* gene displays a high sequence and structure conservation with the *yellow* gene of *Drosophila melanogaster* and other dipterans (Figure S4). Thus, we hypothesized that *ChY* loss-of-function would result in the non-lethal brown* body* (*bwb*) mutant phenotype, as described for the house fly, *Musca domestica* (Heinze *et al.* 2017). Fully–developed *bwb* adults are expected to lack normal melanization, showing a noticeable brownish cuticle colored body, which in turn would be a simple phenotype to detect and score successful Cas9-mediated mutagenesis in *C. hominivorax*. In this context, two sgRNAs targeting the second exon of the *ChY* (Figure 1A) were separately mixed with a high concentration of Cas9 purified protein (500 ng/µl) to form ribonucleoprotein complexes (RNPs). Pre-assembled RNPs were subsequently mixed together and co-injected into 280 early syncytial screwworm embryos. In total, 38 adults were obtained (surviving rate: 13.6%), of which 26 showed lighter pigmented cuticle in a mosaic manner (9 ♂ and 17 ♀; mutagenesis rate: 68.4%; Figure 1B, second column). Since the *bwb*-mutation is recessive and autosomal in *C. hominivorax* (see below), only mutations in both copies of *ChY* will result in the lighter pigmented body phenotype. Therefore, the results also revealed that the CRISPR/Cas9 protocol developed here is capable of inducing somatic biallelic lesions in a large number of individuals, with the vast majority of the mutated cells visible in the posterior (abdomen) and some at the anterior tissues (thorax and legs) of the G_0_ flies (Figure 1B).

While somatic mutants can be directly used for reverse genetic studies, germline gene editing is essential to establish stable mutant lines. To evaluate the inheritance capability of the Cas9-induced mutations in the *ChY* gene, nine G_0_ mosaics were individually inbred (each crossing consisted of 1 *bwb* G_0_ ♂ X 1 - 2 *bwb* G_0_ ♀). Out of the nine putative founder crossings, only two resulted in viable eggs, indicating that the microinjection process might have caused some damage to the precursors of the reproductive tissues during the development of the embryos. Additionally, all the 194 screened G_1_ flies were phenotypically wt, suggesting that these individuals were monoallelic mutants for *bwb*. In order to obtain *ChY* knockout strains (*e.g.*, flies carrying *bwb*-mutations in both copies of the gene) all obtained G_1_ flies were allowed to interbreed freely in cages (massive sibling crossing). The *bwb* mutant phenotype was observed in newly emerged G_2_ individuals, revealing that Cas9-induced mutations are inherited in screwworm. The newly emerged *bwb* mutants were visually identified by the presence of a fully metallic yellowish colored cuticle (Figure 1B, third column), which becomes brownish some hours after the emergence (Figure 1B, fourth column), presumable due to the activity of other genes in the pigmentation pathway (Massey and Wittkopp 2016). A group of 52 flies (29 ♂ and 23 ♀) was selected to start a mutant strain colony in the laboratory (Figure S2).

Total DNA extractions were performed for the G_0_ flies that produced viable eggs after crossings. We confirmed that the *bwb* mosaicism observed in these founders (*e.g.*, flies that produced viable eggs) was caused by the loss-of-function mutations in the *yellow* gene, by performing a T7 endonuclease 1 assay (T7EN1), which revealed the presence of indels at the specific Cas9 targeted sites within *ChY* (Figure S5). Subsequently, the same PCR amplicons were pooled, TA-cloned and Sanger sequenced (*n* = 34 clones) in order to sample the allele variants introduced by Cas9 mutagenesis. A diversity of mutated allele sequences was recovered (Figure 1C and Figure S6), including variants exhibiting small (spanning from ∆ -3 to -13 bp), medium (∆ -50 to -67 bp), and a considerable number of large deletions between both sgRNAs (∆ -360 to -423 bp in 29.4% of the sequences). The putative mutation ratio at G_0_ was found to be 70.6%, as given by CrispRVariants analysis. Finally, we randomly selected and inbred a *bwb*-mutant strain among the multiple lines generated during this experiment, carrying mutant alleles with a -7 bp and -1 bp deletions at the sgR-Y-E2-01 targeted site (Figure S2). This newly created heteroallelic mutant strain was therefore named *ChYellow*^07/01^, which has been maintained for more than 15 generations (phenotype shown in Figure 1D). These results demonstrate that mutations introduced by CRISPR technology are highly stable in *C*. *hominivorax*.

### Cas9-induced somatic and inheritable brown body mutation in the yellow locus of L. cuprina

**Figure 2:**
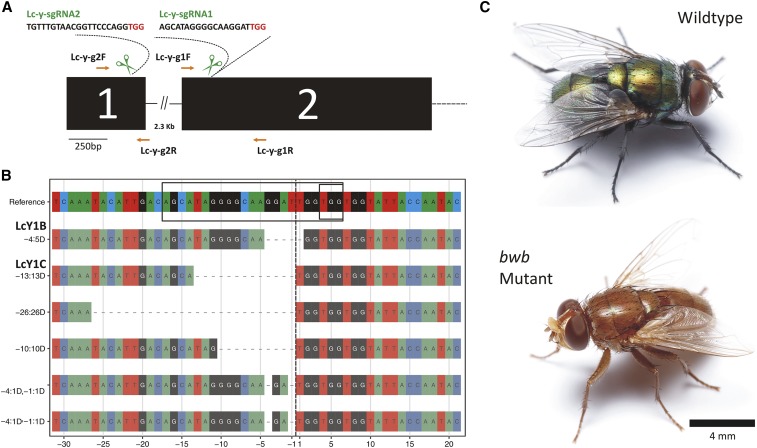
CRISPR/Cas9 targeting of the *yellow* loci (*LcY*) of the blowfly *Lucilia cuprina*. (A) Schematic of the 5′-proximal gene organization of *LcY* and the CRISPR/Cas9 strategy used for knockouts. (B) Alignment of *LcY* variant alleles isolated from G_1_ *bwb*-mutant flies in comparison with the wt (reference) sequence. Targeted site and PAM motif are boxed. Predicted Cas9 cut sites are indicated by vertical black dashed line. Only sequences obtained for Lc-y-sgRNA1 are shown, as it was the only one that demonstrated activity. The homozygous *bwb*-mutant strains LcY1B (-5 bp) and LcY1C (-13 bp) were established after crossing back to the wt parental LA07 strain (Figure S3), and their mutated alleles are shown in the alignment. (C) Comparison between wt (above) and the *yellow* homozygous knockout (below) phenotypes of *L*. *cuprina*.

Initial experiments with *L. cuprina* were performed by injecting embryos with a mix of two sgRNAs targeting the *yellow* gene (*LcY*) and a capped and polyadenylated RNA for Cas9. We had previously used a similar approach to obtain loss of function mutations in the *white* gene of *Drosophila suzukii* (Li and Scott 2016). The designed sgRNAs targeted conserved sequences in the first and second exon of the *LcY* gene (Figure 2A). Of the 127 G_0_ adults that developed from the injected embryos, none showed any loss of pigmentation, suggesting low Cas9 activity or no activity of the sgRNAs. The G_0_ adults were then interbred and, from their offspring, 4 adults were obtained that showed the lightly pigmented *bwb* phenotype. To establish lines, individual *bwb* G_1_ flies were crossed with the parental LA07 strain (Figure S3). After crossing, to determine the nature of the mutations in the G_1_ *bwb* flies, PCR amplicons that spanned the two CRISPR/Cas9 target sites were cloned and sequenced. Several deletion mutations were identified at the location of the sgRNA for exon 2, but no modifications were detected at the predicted cut site for the exon 1 sgRNA (Figure 2B), which suggested that the Lc-Y-sgRNA2 was not as efficient as the sgRNA targeting exon 2 (Lc-Y-sgRNA1). Since the *bwb* G_1_ likely contained two different mutations at the *LcY* locus, strains homozygous for a single mutation were obtained through crossing single G_2_ males with wt females (see methods and Figure S3 for a crossing scheme). The homozygous LcY1B and LcY1C strains (see phenotype in Figure 2C) carry a -5 bp and -13 bp deletion, respectively (showed in Figure 2B). As observed for *C. hominivorax*, the cuticle of *L. cuprina bwb* mutant strains darkens within a few hours of emergence. However, older *bwb* mutants of both species can be differentiated from their respective wt strains on the basis of more lightly pigmented wings and legs.

Next, *L. cuprina* embryos were injected with Cas9-protein loaded with Lc-y-sgRNA1, since this approach efficiently induced mutations in the *C. hominivorax yellow* locus. To increase the chance of detecting a visible phenotype in G_0_ adults, we injected embryos from a cross of wt and the established LcY1B mutant strain (above), as all offspring will be heterozygotes and thus successful knockout of a single *LcY* allele will result in the unpigmented phenotype. Of the 38 adults that developed from injected embryos, 26 (68%) showed a visible *bwb* phenotype as shown by patches of lightly pigmented bodies (Figure S3). Subsequently, we injected embryos (*n* = 115) from the wt strain, with the same Cas9:Lc-y-sgRNA1 pre-assembled RNP, to evaluate the frequency of biallelic hits. Of the 35 adults that developed from the injected embryos (surviving rate: 30.4%), 19 showed patches of lightly pigmented cuticle (mutagenesis rate: 54%). As seen with *C. hominivorax*, injecting *L*. *cuprina* embryos with Cas9 protein preloaded with sgRNAs appears to be a particularly efficient method for obtaining mutations at the targeted gene.

### Mutational inheritance is dependent on Cas9 and sgRNA concentration

We then asked if CRISPR/Cas9 genome editing efficiency is modulated by the final concentration of the delivered Cas9 RNP complexes into the screwworm embryos. To address this, *C. hominivorax* embryos were injected with a high (500 ng/µl) and a lower (360 ng/µl) concentration of Cas9 protein pre-assembled with sgRNAs against the *ChY* gene (Table 1). A single microinjector operator carried out all experiments using the same injection procedure in order to ensure the reproducibility of the technique. A lower proportion of injected embryos developed into adults with the high Cas9 concentration (9.5%; *n* = 38/400) compared with the lower concentration (16.9%; *n* = 63/373), or buffer alone (19.4%; *n* = 62/320), which suggested that the Cas9 protein may have some toxic effects at high concentrations. However, a positive relationship between the RNP concentration used and the mutagenesis rate was obtained as the proportion of flies with a mosaic *bwb* phenotype increased from 55.6% (*n* = 35/63) with the lower concentration to 73.7% (*n* = 28/38) with the higher Cas9 RNP concentration (Table 1). Furthermore, flies that developed from the embryos injected with high Cas9 RNP concentration frequently showed larger body areas of low pigmentation (Figure S7). This observation indicated that biallelic mutations are favored when higher concentrations of RNP are used.

**Table 1:** Effect of CRISPR/Cas9 RNPs concentration on the screwworm survival, mutagenesis and germline transmission

Cas9 (ng/µl)	sgRNA (ng/µl)	Injected embryos	Hatched Larvae^a^	Pupae^a^	Adult Survivors at G_0_^a^			*bwb* mosaics at G_0_^b^			*bwb* transmission to G_1_ (1 ♂ G_0_*^mosaic^* x 2 ♀ *bwb*)^d^	
					♀	♂	Total	♀	♂	Total	Founders^c^	Inheritance^e^
Uninjected	Uninjected	427	323 (75.6%)	157 (36.8%)	76 (17.8%)	67 (15.7%)	143 (33.5%)	0	0	0	—	—
Mock	Mock	320	192 (60.0%)	71 (22.2%)	29 (9.1%)	33 (10.3%)	62 (19.4%)	0	0	0	—	—
360	145	373	180 (48.2%)	82 (21.9%)	29 (7.8%)	34 (9.1%)	63 (16.9%)	15 (23.8%)	20 (31.7%)	35 (55.6%)	8/10 (80%)	38.4 ± 4.3%
500	200	400	137 (34.2%)	48 (12.0%)	23 (5.75%)	15 (3.75%)	38 (9.5%)	18 (47.4%)	10 (26.3%)	28 (73.7%)	6/8 (75%)	81.9 ± 14%

**a**, percentage relative to the number of embryos; **b**, percentage relative to the number of adult survivors; **c**, proportion of G_0_ *bwb* mosaic males that transmitted Cas9-derived mutant alleles to the G_1_ generation (founders) after crossing with *bwb* females from the *ChYellow* ^07/01^ mutant strain; **d**. crosses made to evaluate new mutated alleles for complementation allowing to estimate germline transmission; **e**, average ± SEM of G_1_ biallelic *bwb* mutants obtained from crossing G_0_ mosaic males and *bwb* females (individual result from each cross are shown in Table S3);

Since embryos were injected at the posterior end prior to pole cell formation, it was anticipated that this would lead to mutations of the *ChY* locus in the germline. In order to estimate the level of induced germline mutagenesis, mosaic G_0_ males were crossed with virgin *bwb* females, from the earlier established *ChYellow*^07/01^ strain, and their offspring screened for the brown* body* phenotype. The proportion of G_0_ males that produced *bwb* offspring was similar for the high (6 of 8 fertile males) and low (8 of 10) Cas9 concentration experiments (Table 1, Table S3). However, from the crosses with the G_0_ males using the lower Cas9 concentration injections, 18.5–49.3% (Avg. ± SEM; 38.4 ± 4.3%) of the offspring had a brown* body* phenotype. Flies derived from the high concentration experiment produced *bwb* mutant progeny at a higher frequency ranging from 13 to 100% (81.9 ± 14%; Table 1 and Table S3). Hence, it appears that injecting embryos with a high Cas9 concentration led to a higher frequency of germline mutations at the targeted locus.

### Disruption of transformer gene causes masculinization of XX screwworm flies

*Transformer* (*tra*) is the master gene that controls sex determination in many Diptera species, including blowflies (Scott *et al.* 2014b). Briefly, *tra* transcripts in *C*. *hominivorax* are sex-specifically spliced such that only the female variant (*tra*^F^) encodes a full-length functional TRA protein, which triggers the female development pathway. The longer transcript found in males contain an additional exon with in-frame translation stop codons and as a consequence encodes a short protein that is thought to be non-functional (Li *et al.* 2013). The importance of *tra* for female development was demonstrated by RNA interference (RNAi) experiments in *L. cuprina*, *Lucilia sericata* and *Cochliomyia macellaria* (Concha and Scott 2009; Li *et al.*2013). Briefly, injection of *tra* double-stranded RNA into the posterior end of precellular embryos led to XX individuals developing external male genitalia and testes-like structures.

**Figure 3:**
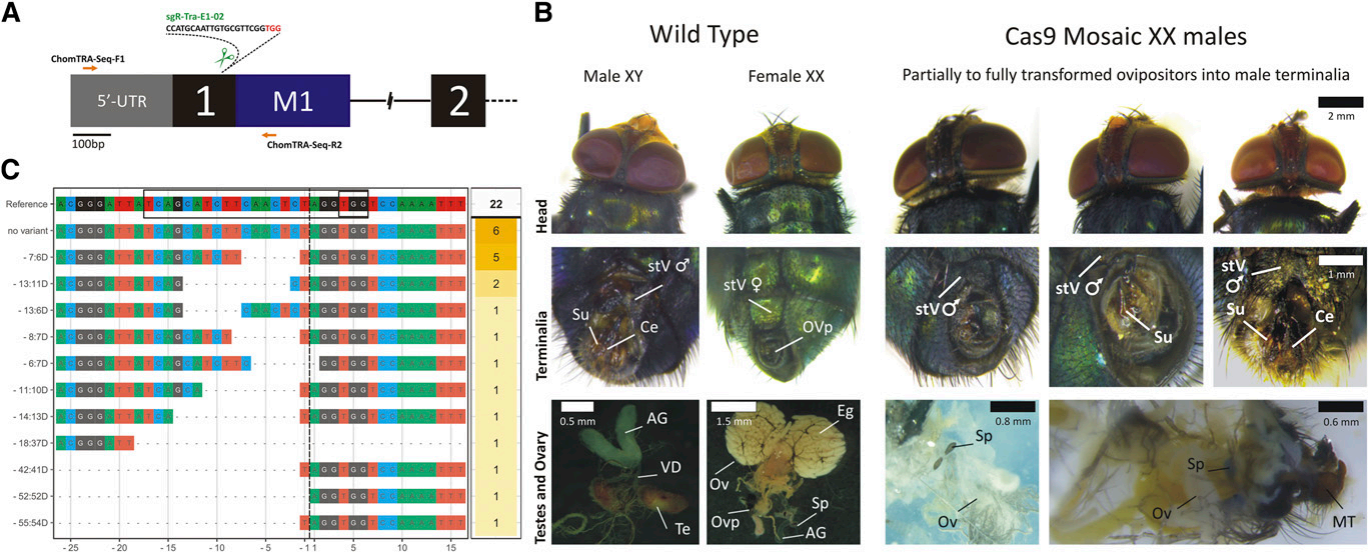
Knockout of screwworm *tra* loci (*Chtra*) causes masculinization of XX flies. (A) Schematic of the 5′-proximal *Chtra* gene organization and CRISPR strategy used for knockouts. (B) Intersexed phenotypes of *Chtra* G_0_ mosaic mutants (leftmost) in comparison with wt male and female (rightmost). Adult flies head (above), genitalia (middle) and dissected reproductive tissue (below) are shown. Cas9-disruption of *Chtra* gene leads to the development of abnormal genitalia and ovaries in G_0_ females, ranging from partially to fully transformed ovipositors into male terminalia. (C) CrispRVariants (Lindsay *et al.* 2016) plots of *Chtra* Cas9-induced mutant alleles found in G_0_ flies in comparison with the wt sequence. Vertical black dashed line indicates the predicted position of Cas9 cut site (see Figure S8 for a more comprehensive alignment). Number of sequenced clones per allele is shown in the right yellow box. Abbreviations used: stV, sternite V; Su, sustylus, Ce, cerci; AG, accessory gland; VD, vans deferens; Te, testis; MT, male terminalia; OVp, ovipositor; OV, ovary; Eg, eggs, and; Sp, spermathecal (as described by Spradbery and Sands 1976).

In this context, we targeted *Chtra* to determine if the CRISPR protocol established for *ChY* could be used for efficient Cas9-mediated gene disruption of another locus in the screwworm genome. We carried out microinjections in *C. hominivorax* embryos targeting the exon-1-intron-1 boundary region of *Chtra* gene (Figure 3A). In total, 24 viable adults developed from 260 injected embryos (9.2% survival, similar to our previous results with *ChY*), of which 14 were phenotypically normal males. Out of the 10 surviving G_0_ females, four showed normal female characteristics while six (60%) developed intersexual phenotypes that were characterized by the presence of an abnormal terminalia with male-like structures, but a female interocular width (Figure 3B). The intersex phenotype appeared as partially to fully transformed ovipositors into male genitalia and, upon dissection, these flies also exhibited abnormal reproductive tissues, including the absence of female structures and/or atypical ovary development (Figure 3B, last row of the panel). To confirm that the male-like structures found in these G_0_ females were due to somatic mutations in the *Chtra* gene, 3 mosaic flies showing different degrees of transformation were selected (intersex individuals showed in Figure 3B), DNA extracted and analyzed by T7EN1 assay (Figure S5). Results showed band migration patterns consistent with the expected Cas9 cleavage from the experimental design, while no activity in the control wt fly was observed, revealing that these individuals carried indels at the targeted region of *Chtra*. The spectrum of mutated alleles induced by Cas9 was then accessed via Sanger sequencing of the same PCR amplifications used in the T7EN1 assay (Figure 3C and Figure S8). Sequenced clones (*n* = 22) confirmed the presence of indels in *Chtra* gene, which were mostly small deletions (∆ -6 to -13 bp), although some medium deletions (∆ -37 to -54 bp) were also found. The mutagenesis efficiency at G_0_ was predicted to be 72.7%, as given by CrispRVariants analysis.

## Discussion

Developing efficient gene manipulation strategies in non-model insects is still a challenge that slows the investigations on their unique biology and natural adaptations. Additionally, the implementation of new genetic strategies to manage economically and medically important insect pest species also requires the development of efficient genome manipulation tools and protocols. In the present study, we described CRISPR/Cas9 site-directed and heritable mutagenesis protocols for *in vivo* functional analysis of candidate loci in the screwworm fly, *C. hominivorax*, and the Australian sheep blowfly, *L. cuprina*. To our knowledge, this is the first demonstration of a directed mutagenesis induced by CRISPR/Cas9 in *Cochliomyia*. The methods serve as the basis for further functional genomic studies in *C*. *hominivorax*, as well in other calliphorids and related biological systems.

Until now, reverse genetic studies in calliphorids have been performed by RNAi-mediated loss-of-function experiments (Mellenthin *et al.*, 2006; Concha and Scott 2009; Li *et al.* 2013), while homozygous transgenic strains have been developed by *piggyBac*-mediated germline transformation (Li *et al.* 2014; Yan and Scott 2015; Concha *et al.* 2016). Although these resources provided means to functionally investigate genes of interest in these species, they have limitations that CRISPR/Cas9 genome editing can help to address. For example, directed injection of double-stranded RNA (dsRNA) into embryos is prone to affect several off-target transcripts (Smith *et al.* 2017) and its effects are transient unless stabilized as part of a transgenic hairpin construct (Brown *et al.* 2003). On the other hand, CRISPR-mediated genome editing usually generates stronger and more consistent phenotypes and is less prone to off-target effects (Evers *et al.* 2016; Housden and Perrimon 2016). In contrast to RNAi, genomic modifications by *piggyBac* are heritable, but often display a lower frequency of germline transformation in comparison with CRISPR induced knockouts. Another concern of transposon-mediated transformations is that the transgene is randomly integrated into the genome, which in turn requires individual strains to be evaluated with respect to their fitness. As genome editing by CRISPR is site-directed, it offers the opportunity to decide where the edits will be performed in the genome. Furthermore, in the case of transgenic sexing strains designed for use in potential SIT programs, it is often desirable to stabilize the genetic construct in the genome of the insects by removing one of the *piggyBac* arms, to prevent remobilization of the transgene (Schetelig *et al.* PNAS 2009). The potential use of CRISPR to create transgenic lines by performing directed knock-ins would be helpful to avoid this technical issue. Here, we reported a high frequency of CRISPR/Cas9-meditated mutagenesis of the *ChY*, *LcY*, and *Chtra* genes, particularly at the high concentration of Cas9/sgRNA complex. Furthermore, a high proportion of surviving G_0_ showed a mosaic phenotype, suggesting that mutations had been introduced at both copies of the targeted genes. CRISPR/Cas9 G_0_ mosaic mutants provide the benefit of allowing the functional study of genes that are essential for development or normal cell function, for which a homozygous knockout line would be lethal.

We found that embryonic injection with a high concentration of Cas9 protein (450 to 500 ng/µl) preloaded with sgRNAs promotes a high mutagenesis frequency at the *yellow* locus in *C. hominivorax* and *L. cuprina*. However, the survival frequency of injected embryos was slightly lower with the high concentration of Cas9, as also observed for other insect species (Kistler *et al.* 2015; Li *et al.* 2017b, 2017a). Hence, we believe that for most targets a mid-range concentration (400 ng/µl) would be suitable to balance survival rate and mutagenesis efficiency, despite the need of increasing the number of injected embryos and replicates for statistical comparisons. A high Cas9 concentration might be desirable when targeting recessive autosomal markers, as it favors biallelic hits and thus a mutant phenotype in the surviving G_0_. The need for Cas9 protein could be avoided by making transgenic strains that express Cas9 either in the germline for generating stable mutant lines or in all cells for screening G_0_ for a mutant phenotype. For example, *Drosophila* strains that produce Cas9 in the germline have been used for efficient gene editing (Kondo and Ueda 2013; Ren *et al.* 2013; Port *et al.* 2015). In addition to generating random indels at the targeted gene as shown in this study, specific nucleotide changes can be made if repair is mediated by the homology-directed repair (HDR) mechanism with a supplied repair template. For example, Aumann *et al.* (2018) recently used short single-stranded DNA donor (ssODN) to introduce point mutations in the genome of the Mediterranean fruit fly, *Ceratitis capitata*. The authors described a highly efficient protocol for CRISPR knockin in this invasive insect pest, where ∼86% of G_0_ fertile females produced mutant offspring and transmitted the edited allele to 71 to 79% of their progeny. Therefore, a logical next step would be to establish this approach in *L. cuprina* and *C. hominivorax* in order to promote specific changes to genes of interest. This could be done, for instance, to delete TRA/TRA2 binding sites that are hypothesized to be important for sex-specific splicing of *tra* transcripts in blowflies (Concha and Scott 2009; Li *et al.* 2013).

Currently, the only markers available for germline transformation of *L. cuprina* and *C. hominvorax* are fluorescent protein genes driven by a strong constitutive promoter (Concha *et al.* 2011, Concha *et al.* 2016). While they greatly facilitate identification of transgenic flies, the markers may not be appropriate if an experiment requires low-level background fluorescence. For such experiments the *bwb* mutant strains developed in this study could be useful recipients for germline transformation with vectors containing *LcY*^+^ or *ChY*^+^ as the marker gene. Transgenic flies would be easily identified through rescue of the wt body color.

We further demonstrated that the CRISPR protocol developed for the *yellow* locus is suitable for generating knockouts in other regions of the screwworm genome, by targeting *transformer* (*tra*; Figure 3A). This is a key gene required for normal female development in many dipterans (Schutt and Nothiger 2000; Verhulst *et al.* 2010). Therefore, we hypothesized that Cas9-induced frameshifts in the *C. hominivorax tra* gene would lead to masculinization of G_0_ females, which is similar to what was observed with RNAi knockdown experiments in the closely related species *C. macellaria* (Li *et al.* 2013). We found that Cas9-mediated mutagenesis of the exon1-intron1 boundary of *Chtra* led to the development of intersexual flies showing a normal female interocular width, but with anomalies in their genitalia and reproductive tissues that were male-like (Figure 3B). Overall, out of 10 surviving females, obtained after Cas9-sgRNA injections, six presented different degrees of masculinization, including a single female with a fully developed male terminalia (Figure 3B, last column of the panel). All adult males developed from injected embryos showed normal phenotypes, suggesting that mutations in *Chtra* are not hazardous for male development. However, it is important to note that any fully transformed XX males would be undetectable in our results due to the current lack of sex-linked markers for *C*. *hominivorax*. As expected by its high conservation between closely related species, the results confirmed that *tra* is required for normal female development in *C*. *hominivorax*, and suggested that the *Chtra* or *Lctra* genes could be potential targets for future Cas9-based systems for genetic control of these pests. For example, CRISPR-based gene drive strains could be evaluated in the future for population suppression of these important pests of livestock (Hammond *et al.* 2016; Scott *et al.* 2018; Kyrou *et al.* 2018). Concha and Scott (2009) found that some fully transformed XX *L. cuprina* flies are fertile, as it was also described by Zhao *et al.* (2019) for the Oriental fruit fly, *Bactrocera dorsalis*. Thus both XY and fully transformed XX flies could contribute to population suppression. In addition to being a target for a “homing” gene drive system, targeting Cas9 to the *tra* locus in *L. cuprina* and *C hominivorax* could be an effective means for producing only males for a more efficient SIT program (Kandul *et al.* 2019). Rendón *et al.* (2004) observed that the field release of male sterilized medflies was several-fold more effective than bisexual releases. Therefore, for a potential sterile male field release, males and intersexes could be generated by crossing strains that express Cas9 in somatic cells and sgRNAs constitutively. The intersexes would be expected to be sterile. A disadvantage of this approach is that a large number of virgin females would need to be collected from one strain to set the cross, which could be impractical in a production facility. An alternative would be to regulate Cas9 expression using the tetracycline transactivator (tTA), which is easily repressed by adding tetracycline to the diet. This would be similar to the previously generated tetracycline repressible female lethal strains (Li *et al.* 2014, Concha *et al.* 2016, Yan and Scott 2015). In addition to *tra*, it would be desirable to evaluate other sgRNAs against the *doublesex* (*dsx*) and *fruitless* (*fru*) genes, which are also essential for female development (Scott *et al.* 2014b). The highly conserved female-specific exon of *dsx* in particular appears to be a promising target for gene drive (Kyrou *et al.* 2018).

Perhaps one of the most interesting and complex questions in Calliphoridae evolution is how ectoparasitism (obligatory and facultative) arose independently in some lineages of the family, including the ones that led to the *C. hominivorax* and *L*. *cuprina* species (Stevens *et al.* 2006; Hall *et al.* 2016; Junqueira *et al.* 2016). The major transition from a free living to a parasitic organism is expected to require a number of morphological, reproductive, and physiological adaptions. In the case of calliphorids, we are particularly interested in molecular adaptations toward host seeking by chemical signal transduction as chemoreception plays an essential role during specialization, behavior, and niche establishment (Hansson and Stensmyr 2011). In contrast to their closely related cousins that seek dead animals, the parasitic calliphorids must seek out live warm-blooded animals. The availability of assembled genome sequences together with the establishment of the CRISPR technology, described here, provide the foundation for functional investigations on genomic regions linked to the evolution of natural traits in these parasitic flies. This opportunity provides the nexus to interrogate evolutionary aspects of *C*. *hominivorax*, which could translate in the development of new environmentally friendly management strategies for parasitic calliphorids, and possibly for other arthropod parasites and disease vectors.
